# A Scorpion Peptide Exerts Selective Anti-Leukemia Effects Through Disrupting Cell Membranes and Triggering Bax/Bcl-2-Related Apoptosis Pathway

**DOI:** 10.3390/biom15121751

**Published:** 2025-12-18

**Authors:** Xiaoping Dong, Siwei Yi, Yaqin Yang, Yushuo Wang, Lingxiang Wang, Jingjing Huang, Yong Zeng, Zhonghua Liu

**Affiliations:** 1 National & Local Joint Engineering Laboratory of Animal Peptide Drug Development, College of Life Sciences, Hunan Normal University, Changsha 410081, China; 2 Peptide and Small Molecule Drug R&D Platform, Furong Laboratory, Hunan Normal University, Changsha 410081, China; 3 The State Key Laboratory of Developmental Biology of Freshwater Fish, College of Life Science, Hunan Normal University, Changsha 410081, China

**Keywords:** anticancer peptide, MV-4-11, apoptosis, leukemia, membrane disruption

## Abstract

Numerous modern scientific studies have demonstrated that animal venoms harbor a wealth of diverse anticancer active components, serving as a valuable resource for the development of natural antitumor drugs. AI-based computation and prediction models enable rapid screening of extensive active peptides. In this study, the anticancer activity of seven peptides was predicted using our previous deep learning model. Further verification experiments confirmed that Lpep3 can selectively and efficiently inhibit the growth of leukemia cells. Electron microscopy observations revealed cell shrinkage in morphology and honeycomb-like perforations on the cell membrane in the treated group. It is hypothesized that high-concentration peptides disrupt the cell membrane and increase cell permeability, which was confirmed by trypan blue staining and Calcein-AM/PI double-staining assays. Lpep3 induces the release of lactate dehydrogenase (LDH) and ATP in a concentration-dependent manner, further suggesting that this peptide disrupts the cell membrane. In addition, although Lpep3 does not affect the cell cycle of MV-4-11, it can induce cell apoptosis. Western blotting and RT-qPCR results showed that compared with the control group, the expression levels of Bax were upregulated, while the expression level of Bcl-2 protein was downregulated in the Lpep3 group. In vivo experiments demonstrated that Lpep3 has good biological safety, and compared with the control group, the Lpep3 group could inhibit the growth of tumor cells in mice. Collectively, Lpep3 is characterized by high potency and specificity and may serve as a promising lead compound for the development of anti-leukemia drugs.

## 1. Introduction

Cancer remains the leading disease threatening public health. The increasing incidence and mortality of cancer can be attributed to the side effects, drug resistance, and insufficient therapeutic efficacy associated with conventional treatments and therapeutic strategies [1,2]. Exploring more cancer treatment options is urgently needed. Recently, anticancer bioactive peptides (ACPs) derived from animal venoms have emerged as promising therapeutic candidates in the drug discovery field due to their high cell permeability, low toxicity, specificity, and reduced side effects. For example, BMAP-27 and BMAP-28 are both antimicrobial peptides (AMPs) derived from bovine cathelicidin and have been demonstrated to exert antitumor activity against leukemia [3]. In addition, P18 has been reported to selectively inhibit multiple human cancer cells, including Jurkat, K562, and MDA-MB-361 [4]. Melittin is a 26-amino acid residue peptide isolated from the venom of the European honeybee Apis mellifera. It has also been found to exert diverse anticancer effects by inhibiting cell proliferation, inducing apoptosis, and triggering direct necrosis [5]. In addition, there are numerous toxins or peptides derived from different scorpion species that inhibit tumor proliferation, invasion, and metastasis, indicating their innovative application potential in cancer therapy. For instance, CTX was reported to inhibit the migration and invasion of glioma cells by targeting chloride channels and matrix metalloproteinase 2 [6]. BmK AGAP and BmK AGAP-SYPU2, isolated from the Chinese scorpion *Mesobuthus martensii*, exhibited significant anticancer activity by suppressing the epithelial–mesenchymal transition (EMT) and migration of human breast cancer [7,8].

Anticancer peptides (ACPs) represent one of the most promising classes of therapeutic agents for the treatment of common human cancers. Currently, the primary advantages driving the development of most ACP-based drugs include their amphipathic structure, cationic nature, membrane interaction capacity, broad-spectrum activity, low tendency to induce drug resistance, and low immunogenicity [9,10]. Numerous ACPs have obtained FDA approval or advanced to late-stage clinical trials, and considerable progress has been achieved in their clinical translation [11,12,13]. However, the journey of exploration remains protracted for ACP candidate drug development [14]. For instance, the high proteolytic susceptibility of natural peptides causes short half-lives and poor in vivo bioavailability [15]. Moreover, the inherent conformational properties of ACPs impair their functionality in complex physiological milieus, while low membrane permeability limits the interaction of certain ACPs with intracellular targets [16]. Furthermore, the emergence of a certain degree of drug resistance in some ACPs further contributes to suboptimal therapeutic outcomes [17].

In terms of methodologies for anticancer peptide discovery, traditional wet experiments for verifying peptide anticancer activity are both time-consuming and costly. Consequently, researchers have increasingly developed computational methods for ACP prediction based on deep representation learning features using computer-aided machine learning [18]. These approaches not only facilitate high-throughput ACP design and screening, but also conserve human and material resources, thereby providing a novel direction for anticancer drug development. Numerous studies have demonstrated that AI-derived prediction can successfully identify novel antimicrobial and anticancer peptides, thereby paving new avenues for accelerating drug discovery [19,20,21,22].

In this study, we downloaded venom animal toxin peptides from public databases and used an anticancer model, CNBT-ACPred, established by deep learning algorithms to predict the anticancer activity of these peptides. Based on the prediction scores and species diversity, seven peptides were selected for subsequent activity verification. In vitro experiments confirmed that Lpep3, which has been previously reported to have antibacterial activity, can selectively and effectively inhibit the growth of leukemia cell line MV-4-11 and induce apoptosis. In vivo experiments showed that this peptide has good biosafety and can effectively inhibit tumor growth in mice. In conclusion, compared with previously reported anticancer peptides, the peptide identified in this study exhibits superior targeting ability and stronger activity. Our findings indicate that this scorpion venom-derived peptide may serve as a promising candidate for leukemia therapy, offering a novel perspective on venom-inspired anticancer drug development.

## 2. Materials and Methods

### 2.1. Chemical Synthesis of Peptides

In our previous study, large-scale peptides from diverse species were downloaded. After further processing and filtering, the anticancer activity of the remaining peptides was predicted using our self-constructed anticancer model, CNBT-ACPred [23]. Based on the criterion that the average prediction score is greater than 0.73, 276 peptides derived from toxic animals meet this condition. Further, candidate peptides were selected from this pool and demonstrated strong in silico efficacy potential because of their prediction scores > 0.9. Subsequently, from this high-scoring shortlist, we finalized the peptide selection by maximizing the phylogenetic diversity of their source organisms. This approach prevented the selection of multiple, highly similar peptides from a single phylogenetic clade and enabled us to screen for potential broad-spectrum efficacy across diverse species. From the perspectives of average prediction score and species diversity, seven peptides were chosen for synthesis and activity verification. The peptides were synthesized on a Rink amide-AM resin using the SPPS (solid-phase peptide synthesis) method, as previously described by Nanjing peptide Biotech Ltd. (Nanjing, China) [24]. The purity or identity characterization of the synthesized peptides is provided in Supplementary Materials.

### 2.2. Cell Lines and Cell Culture

All cell lines, including CT26.WT, LLC, MV-4-11, HCT-116, ID8, Jurkat, L1210, OVCAR-8, YAC-1, MDA-MB-231, MEL, Mia PaCa-2, B16-F10, HepG2, A549 Hela, 4T1, Panco2, K562, RAJI, A375, HL-60, H1975, HaCat, HUVEC, and HDF, were obtained from the National Collection of Authenticated Cell Cultures (Shanghai, China) or saved in the lab and cultured in RPMI-1640 or DMEM or IMEM medium supplemented with 10% (*v*/*v*) fetal bovine serum (FBS), 100 units/mL penicillin, 100 µg/mL streptomycin, and 0.05% (*v*/*v*) L-glutamine serum in a humidified atmosphere of 5% CO_2_ at 37 °C.

### 2.3. Isolation of Peripheral Blood Mononuclear Cells (PBMCs)

Briefly, fresh anticoagulated whole blood was collected and diluted with 1× PBS buffer at a ratio of 1:1, followed by gentle mixing. Then, an appropriate volume of mononuclear cell separation medium was added to a sterile centrifuge tube, and the diluted blood sample was carefully layered onto the surface of the separation medium (separation medium–diluted whole blood = 1:2) while maintaining a clear interface between the two layers. Then, it was centrifuged at 450× *g* for 20–30 min at room temperature. After centrifugation, the plasma layer was aspirated and discarded, then the PBMC layer was carefully collected (i.e., the buffy coat) and transferred to a 15 mL centrifuge tube. The cells were resuspended by adding 10 mL of 1× PBS buffer to the centrifuge tube, centrifuging at 400× *g* for 10 min at room temperature, and discarding the supernatant. This step was repeated 1–2 times. The isolated PBMCs were then ready for subsequent experiments.

### 2.4. Cell Viability Assays

To determine the effects of Lpep3 on cell cytotoxicity, the Cell Counting Kit-8 (CCK8) assay was performed according to the manufacturer’s protocol. Cells were seeded at 8 × 10^3^ cells per well and treated with Lpep3 at different concentrations (100, 50, 25, 12.5, 6.25, 3.125, 1.56, 0.78, 0.39 μM) for 24 h. Subsequently, 10 μL of CCK8 reagent (Abbkine, Wuhan, China) was added to each well and incubated for 1–4 h at 37 °C. The absorbance at 450 nm was measured using a microplate reader (BioTek, Winooski, VT, USA). The half-maximal inhibitory concentration (IC_50_) was defined as the peptide concentration required to reduce cell viability by 50% relative to the untreated control. IC_50_ values were calculated using non-linear regression analysis of the dose–response curves in GraphPad Prism (8.0.2). The percentage of cellular viability was calculated using the following equation:

$$Cell\;Viability\;\left(\%\right)=\frac{OD_{Sample}-OD_{Blank}}{OD_{Control}-OD_{Blank}}\times 100\%$$

### 2.5. LDH Leakage Assay

According to the manufacturer’s instructions, the LDH release assay was used to determine the integrity of the membrane. In brief, MV-4-11 cells were seeded and cultured in a 96-well cell culture plate (1 × 10^4^ cells/well) and treated with different concentrations (0.039, 0.156, 0.625, 2.5 μM) of Lpep3 for different times. After treatment, 50 μL of culture medium was collected and mixed with the reaction mixture (50 µL) and incubated at room temperature for 30 min, followed by stopping with 50 µL of the stop solution. The absorbance was read using a microplate reader at 450 nm.

### 2.6. Flow Cytometric Analysis of Apoptosis and Cell Cycle

For the cell apoptosis assay, MV-4-11 cells were treated with 0.5 and 1 µM Lpep3 for 24 h; no drug treatment was viewed as the control. According to the Annexin V-EGFP/PI kit’s instructions (Keygene Biotech, Nanjing, China), cells were harvested and stained with 5 µL Annexin V–FITC, 5 µL PI, and incubation buffer for 15 min at 37 °C. They were then subjected to flow cytometry to analyze the percentage of apoptotic cells. For the cell cycle assay, cells were treated with the same concentration for 24 h. After that, a total of 3 × 10^5^ cells was collected and fixed in cold 70% ethanol. The plates were finally stained with PI (propidium iodide, 1 mg/mL), TritonX-100 (0.1%), and RNAse (100 mg/mL) and incubated at 37 °C for 20 min in a dark environment. A flow cytometer was employed to identify fluorescence signals. Cell cycle distribution was then determined by flow cytometry (Beckman Coulter, Fullerton, CA, USA).

### 2.7. Calcein/PI Double Staining

MV-4-11 cells were seeded in 24-well plates at 10 × 104 cells per well and cultured at 37 °C and 5% CO_2_. After 24 h, cells were treated with 0.5 and 1 μM Lpep3 for another 24 h. The cell viability was assessed using Calcein-AM and PI according to the manufacturer’s instructions (Beyotime, Beijing, China). Cells were stained with 250 µL of a mixture containing 0.25 µL Calcein-AM and 0.25 µL PI under dark conditions at 37 °C for 10 min and then immediately photographed with a fluorescence microscope system (Olympus, Tokyo, Japan).

### 2.8. Transmission Electron Microscopy (TEM)

For TEM analysis, Lpep3-treated cells were first prefixed with 2.5% glutaraldehyde at room temperature. The cells were then gently scraped off and collected into pellets by centrifugation. The cell pellets were further fixed with glutaraldehyde at 4 °C for 2–4 h, followed by washing with phosphate buffer. Subsequently, the samples were postfixed with 1% osmium tetroxide in phosphate buffer and dehydrated through a graded series of ethanol and acetone. The dehydrated samples were infiltrated with mixtures of acetone and Epon 812 epoxy resin, then embedded in pure resin and polymerized at 60 °C for 48 h. Ultrathin sections (60–80 nm) were prepared, stained with uranyl acetate and lead citrate, and finally observed under a transmission electron microscope (JEM-1400, JEOL, Tokyo, Japan) for image acquisition.

### 2.9. Trypan Blue Staining

At a density of 2 × 10^5^ cells per well, cells were seeded in 6-well plates and treated with 0.5 and 1 μM of Lpep3. After being cultured for 24 h, cells were directly collected and centrifuged at 1000 rpm for 3 min. After discarding the supernatant, an appropriate amount of PBS was added to resuspend the cells and prepare a single-cell suspension. The cell suspension was mixed with 0.4% trypan blue solution (Solarbio, Beijing, China) at a 9:1 ratio, stained for 3 min, and then observed and photographed under a microscope.

### 2.10. ATP Synthesis and Release

The ATP content in the culture medium and cells was assessed using fluorescein luciferase (S0027, Beyotime, Beijing, China). The cell extracts and dilution buffer with luciferase were mixed in 96-well microplates, incubated at room temperature for 3–5 min, and then the chemiluminescent RLU values were measured using a multi-function microplate reader (BioTek, VT, USA).

### 2.11. Western Blot Analysis

Whole-cell extracts were prepared in 1× loading buffer lysis (CWBio, Jiangsu, China), and protein concentration was measured using the Bicinchoninic acid (BCA) Protein Assay Kit (Sangon, Beijing, China). Then, 15 μg of protein from different samples was electrophoresed through 12% sodium dodecyl sulfate–polyacrylamide gels, and the proteins were then transferred onto a polyvinylidene fluoride (PVDF) membrane. Blots were treated with 5% non-fat dried milk and left to incubate with primary antibodies at 4 °C overnight, and then they were incubated with horseradish peroxidase (HRP)-conjugated secondary antibodies for 40 min at room temperature. The primary antibodies included anti-Bax (Proteintech, Wuhan, China) and anti-Bcl-2 (Proteintech, China). Finally, the membrane was detected by Super ECL Detection Reagent ECL (Bioprimacy, Wuhan, China) using a fully automated chemiluminescence image analysis system (Tanon, Shanghai, China). The relative expression of each target band was normalized to the intensity of GAPDH. The normalized data from at least three independent experiments were then calculated as the fold change relative to the control group.

### 2.12. Total RNA Extraction, cDNA Synthesis, and Quantitative RT-PCR Analyses

For total RNA extraction, up to 3  ×  10^6^ cells were harvested, and total RNA was isolated using the SteadyPure RNA Purification Kit (Accurate biology, Changsha, China). For cDNA synthesis, 1 µg of the total RNA was reverse-transcribed with the RevertAid First Strand cDNA Synthesis Kit (Thermo Fisher Scientific, Waltham, MA, USA). The template cDNA was amplified using the primer pairs reported in Table 1. GAPDH was used as an internal reference, and the 2^−△△CT^ method was utilized to ascertain relative expression.

### 2.13. Hemolytic Activity Assay

A total of 500 μL fresh whole blood was collected and diluted with 5 mL PBS and then centrifuged at 1000 rpm for 5 min to remove the plasma. The pellet was washed again by adding PBS to a final volume of approximately 10 mL, followed by centrifuging at 1000 rpm for 5 min. This washing step was repeated until the supernatant became clear. The resulting red blood cell pellet was then resuspended in 10 mL PBS. For the hemolysis assay, 200 μL suspension was mixed with 200 μL Lpep3 with different concentrations (100, 50, 25, 12.5, 6.25, 3.125, 1.57, 0.78, 0.39 μM). The positive control consisted of 200 μL suspension mixed with 200 μL 0.1% Triton X-100, while the negative control contained 200 μL RBC suspension and 200 μL PBS. All mixtures were incubated at 37 °C for 1 h, followed by centrifugation at 12,000 rpm for 5 min. The supernatant was collected and measured at 450 nm. The hemolysis rate was calculated using the following equation:

$$Hemolysis\;rate\left(\%\right)=\frac{OD_{Sample}-OD_{negative}}{OD_{Positive}-OD_{nagative}}\times 100\%$$

### 2.14. Xenograft Using Nude Mice

Healthy male BALB/c nu/nu nude mice (aged 4–5 weeks) were purchased from Slac & Jingda Laboratory Animal Co., Ltd. (Changsha, China). All animal experiments were strictly approved by the Institutional Animal Care and Use Committee (IACUC) of Hunan Normal University, and the Guidelines for the Care and Use of Laboratory Animals issued by the National Institutes of Health (NIH, Bethesda, MD, USA) were strictly adhered to throughout the experiment. MV-4-11 cells (5 × 10^6^ cells resuspended in a 1:1 mixture of Matrigel and PBS) were subcutaneously (s.c.) injected into the right axillary fossa of each mouse. When the tumor volume reached 100–150 mm^3^, all mice were randomly divided into two groups (*n* = 4). Mice in the experimental group were intraperitoneally injected with 50 μL of Lpep3 (10 mg/kg), while those in the control group received an intraperitoneal injection of an equal volume of sterile PBS. The day of the first injection was defined as Day 0. Injections were administered every other day, with a total of 6 administrations. Additionally, the tumor volume and body weight of mice in each group were monitored at regular intervals. After the experiment, major organs were dissected, fixed in 4% paraformaldehyde (PFA), and subjected to histological examination. Major organs were further stained with hematoxylin and eosin (H&E) for pathological analysis.

### 2.15. Blood Biochemistry and Liver/Kidney Function Test

After euthanasia, whole blood was collected and allowed to stand at 4 °C for 1 h and then centrifuged at 3000× *g* for 10 min to obtain serum. Serum biochemical and liver/kidney function parameters, including RBC, NEU, HGB, ALT, AST, BUN, and creatinine, were analyzed using an automated chemistry analyzer (TBA-40FR, Tochigi, Japan) according to the manufacturer’s instructions.

### 2.16. Statistical Analysis

Statistical analysis of the data was performed using GraphPad Prism (8.0.2). Data were presented as mean ± SEM. *p* values were calculated by unpaired two-tailed *t*-tests or one-way ANOVA. Bars with different signs indicate significant differences. * *p* < 0.05, ** *p* < 0.01, *** *p* < 0.001 compared to control.

## 3. Results

### 3.1. Preliminary Screening of Venom-Derived Peptides Against Cancer Cell Lines

Based on the anticancer prediction model CNBT-ACPred established by our team previously, there are 276 toxic animal polypeptides whose average prediction score is greater than 0.73. Then, based on the top prediction scores and species diversity, seven candidate peptides were chosen for subsequent experimental validation (Table 2). Lpep4 was not considered in the experimental verification because of poor solubility in PBS and DMSO.

**Figure 1:**
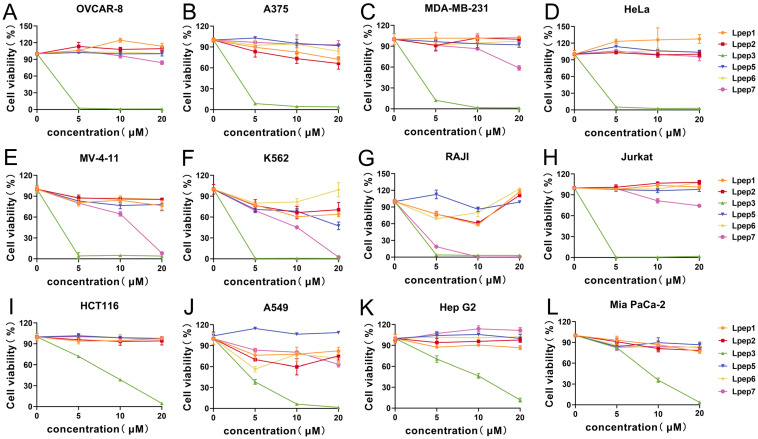
The trends of inhibition rates of 6 candidate peptides against 12 human-derived cancer cell lines at 5 μM, 10 μM, and 20 μM. The tested cell lines include human solid tumors: (**A**) OVCAR-8; (**B**) A375; (**C**) MDA-MB-231; (**D**) HeLa; and human hematological tumors: (**E**) MV-4-11; (**F**) K562; (**G**) Raji; and (**H**) Jurkat. (**I**) HCT116; (**J**) A549; (**K**) Hep G2; (**L**) Mia Paca-2. Each subgraph corresponds to one cancer cell line, with the x-axis representing peptide concentrations and the y-axis representing cell viability rates (%). Different lines indicate different candidate peptides, as labeled in the legend. Data points represent the mean of 3 independent experiments, with error bars indicating the standard error of the mean (S.E.M.).

To comprehensively characterize the anticancer activity of peptides Lpep1–Lpep7, their cytotoxic effects across 24 distinct cancer cell lines were assessed using CCK-8 assays. As shown in Figure 1, Lpep3 emerged as a potent, broad-spectrum cytotoxic peptide. Notably, in OVCAR-8 (ovarian), A375 (melanoma), MDA-MB-231 (breast), HeLa (cervical), MV-4-11 (acute myeloid leukemia), K562 (chronic myeloid leukemia), and Jurkat (acute T-cell leukemia) cells, Lpep3 reduced cell viability to nearly 0% at 5 μM (Figure 1A–H). Even in A549 (lung), HCT116 (colorectal), Mia PaCa-2 (pancreatic), and Hep G2 (liver) cells, which required higher concentrations, Lpep3 still completely suppressed cell growth at 20 μM, underscoring its consistent potency across diverse cancer types (Figure 1I–L). However, other peptides showed minimal cytotoxic effects in most cell lines. For example, in OVCAR-8, A375, MDA-MB-231, HeLa, A549, HCT116, Hep G2, MV-4-11, and Jurkat cells, their treatment (even at 20 μM) maintained cell viability above 90%. While Lpep7 exhibited moderate activity in MV-4-11, K562, and Raji cells, their efficacy was substantially lower than that of Lpep3. In addition, Lpep3 also exhibited superior anticancer activity compared to other peptides in a variety of mouse-derived cancer cell lines (Figure S1). These results indicate that this peptide has a broad-spectrum anticancer potential.

### 3.2. Lpep3 Effectively and Selectively Inhibits the Growth of Leukemia Cells

**Figure 2:**
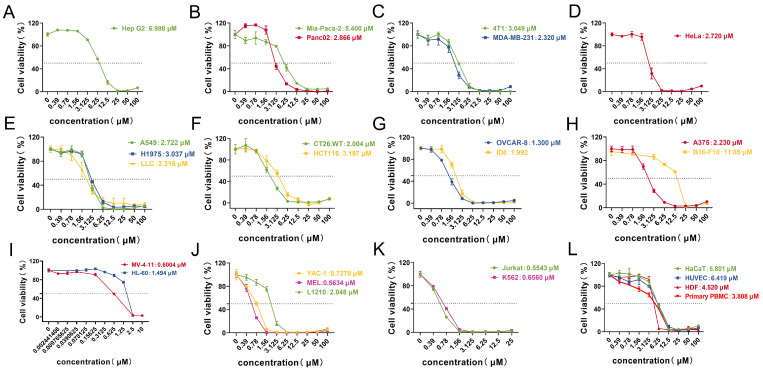
Lpep3 exhibits a potent and selective cytotoxic activity against leukemia cells. The IC_50_ values of Lpep3 in various cancer cell lines grouped by tissue type. (**A**) Hepatocellular carcinoma cells (HepG2); (**B**) pancreatic cancer cells (MIA PaCa-2, PANC02); (**C**) breast cancer cells (4T1, MDA-MB-231); (**D**) cervical cancer cells (HeLa); (**E**) lung cancer cells (A549, H1975, LLC); (**F**) colorectal cancer cells (HCT116, CT26.WT); (**G**) ovarian cancer cells (ID8, OVCAR8); (**H**) melanoma cells (B16-F10, A375). (**I**–**K**) Human hematological tumors (MV-4-11, HL-60, YAC-1, MEL, L1210, K562, and Jurkat). (**L**) Non-cancer cell line and primary PBMCs. Each data point represents the IC_50_ value (μM) calculated from three independent experiments (concentration–response curves), with error bars indicating the standard error of the mean (S.E.M.). IC_50_ values were derived using nonlinear regression analysis of dose–response curves (log (inhibitor) vs. normalized response).

Based on the above results, subsequent studies will focus on determining the IC_50_ of Lpep3 to accurately quantify the intensity of its anticancer activity. The remaining peptides, due to weak or no activity, do not require further in-depth investigation. As shown, in these solid cancers, including hepatocellular (HepG2, Figure 2A), pancreatic (MIA PaCa-2, PANC02, Figure 2B), breast (4T1, MDA-MB-231, Figure 2C), cervical (HeLa, Figure 2D), lung (A549, H1975, LLC, Figure 2E), colorectal (HCT116, CT26.WT, Figure 2F), ovarian (ID8, OVCAR8, Figure 2G), and melanoma (B16-F10, A375, Figure 2H) cells, Lpep3 exhibited IC_50_ values generally within the low micromolar range (≈1.3–11 μM). The ovarian cell was the most sensitive in solid tumor cell lines.

Importantly, Lpep3 showed stronger inhibitory activity on leukemia cells, with IC_50_ values predominantly below 1 μM (Figure 2I–K and Figure S2A). The lowest values approached 0.6 μM in MV-4-11. However, in primary PBMCs, the IC_50_ was 3.808 μM, and in non-cancer cells, the average IC_50_ was approximately 5 μM, which is a nearly 10-fold increase compared to that in cancer cells, providing sufficient evidence for its selectivity (Figure 2L). These results demonstrate that Lpep3 exerts significantly stronger anticancer activity and a higher degree of selectivity toward leukemia cells. Although Lpep3 exhibited strong hemolytic activity, with a 50% hemolysis rate observed at 12.5 μM, the IC_50_ value against leukemia cells was approximately 0.6 μM, representing nearly a 21-fold difference (Figure S2B). Based on this discrepancy, concentrations of 0.5 μM and 1 μM were selected for subsequent experiments.

### 3.3. Lpep3 Induces MV-4-11 Acute Leukemia Cell Death Through Membrane Disruption

**Figure 3:**
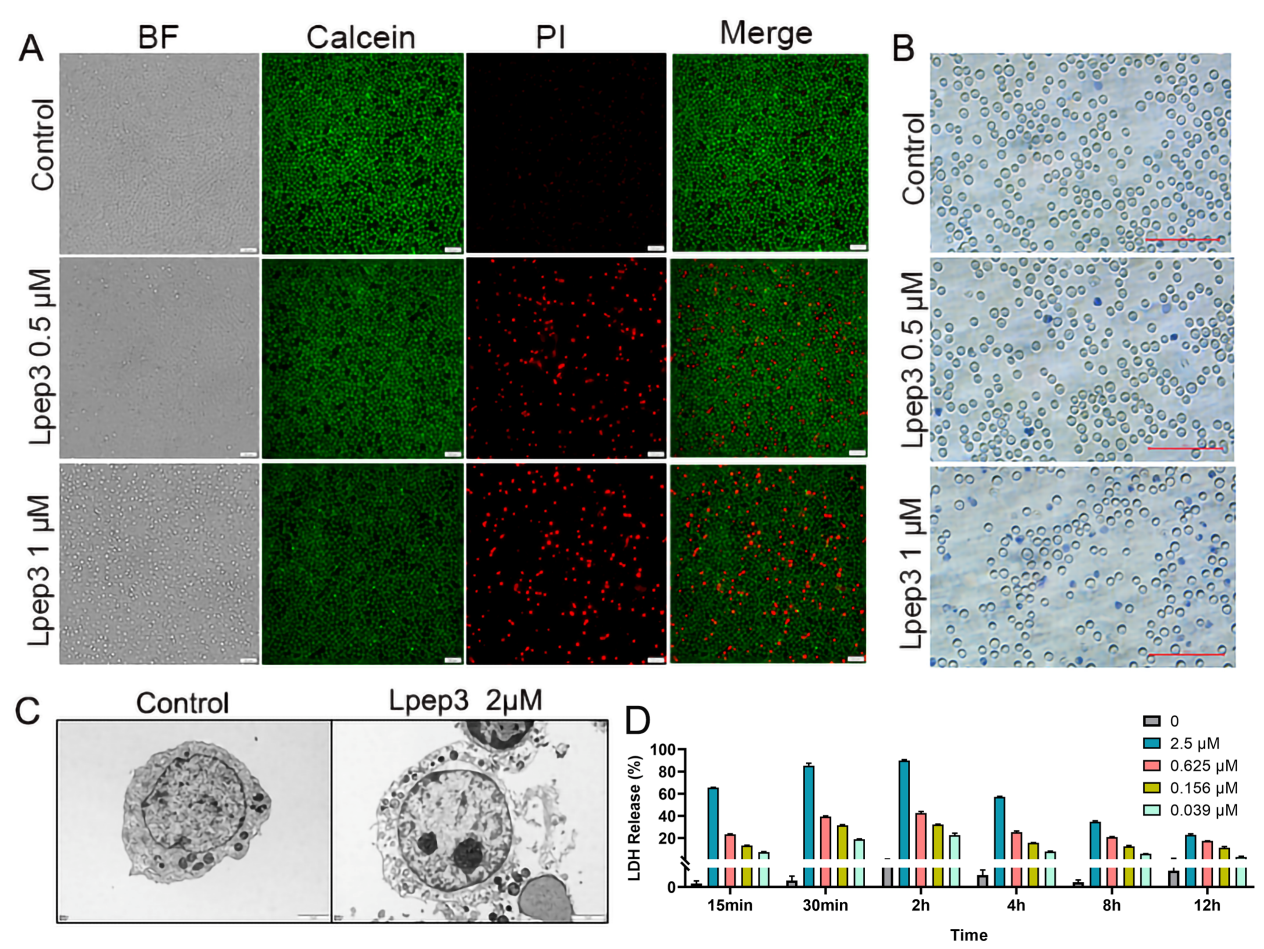
The membrane disruption ability of Lpep3. (**A**) Calcein-AM/PI double staining after MV-4-11 cells were treated with Lpep3 at 0.5 and 1 µM for 24 h. Scale bar is 50 μM. (**B**) Trypan blue staining. Scale bar is 100 μM. (**C**) TEM images of MV-4-11 cells after treatment with 2 µM Lpep3 for 1 h. Scale bar is 5 μM. (**D**) Measurement of LDH leakage after MV-4-11 cells were treated with Lpep3 at different concentrations for 15 min, 30 min, and 2 h.

Based on the IC_50_, MV-4-11 cells, an acute myeloid leukemia (AML) cell line, were selected for subsequent studies. This choice was further motivated by the clinical significance of AML, an aggressive hematologic cancer with limited therapeutic options and frequent drug resistance. Previous studies have shown that the membrane-disrupting effect of anticancer peptides (ACPs) is the main mechanism for inducing cell death. To investigate whether this mechanism applied to our study, TEM, Calcein-AM/PI double staining, and trypan blue staining were conducted. In the untreated group, MV-4-11 cells displayed strong green fluorescence. In contrast, treatment with 1 μM Lpep3 for 24 h resulted in predominant red fluorescence, signifying compromised membrane integrity. This effect was dose-dependent, as fewer PI-positive cells were observed at 0.5 μM (Figure 3A). Consistently, trypan blue staining confirmed a marked increase in membrane-compromised (blue-stained) cells following 1 μM treatment (Figure 3B). TEM was performed to visualize the morphology of MV-4-11 cells after treatment with Lpep3. Compared with the complete cell morphology in the control group, there was obvious leakage of cytoplasmic contents in the cells after treatment with 2 µM for 1 h, indicating damage to the cell membrane (Figure 3C). Moreover, lactate dehydrogenase (LDH) release assays revealed that Lpep3 treatment induced rapid membrane damage, as evidenced by significant LDH release within 15 min. The LDH release rate reached nearly 90% at 2 h. Collectively, these findings demonstrate that Lpep3 compromises MV-4-11 cell membrane integrity in a concentration-dependent manner and rapidly leads to direct cell death.

### 3.4. Lpep3 Induces Apoptosis in MV-4-11 Cells

**Figure 4:**
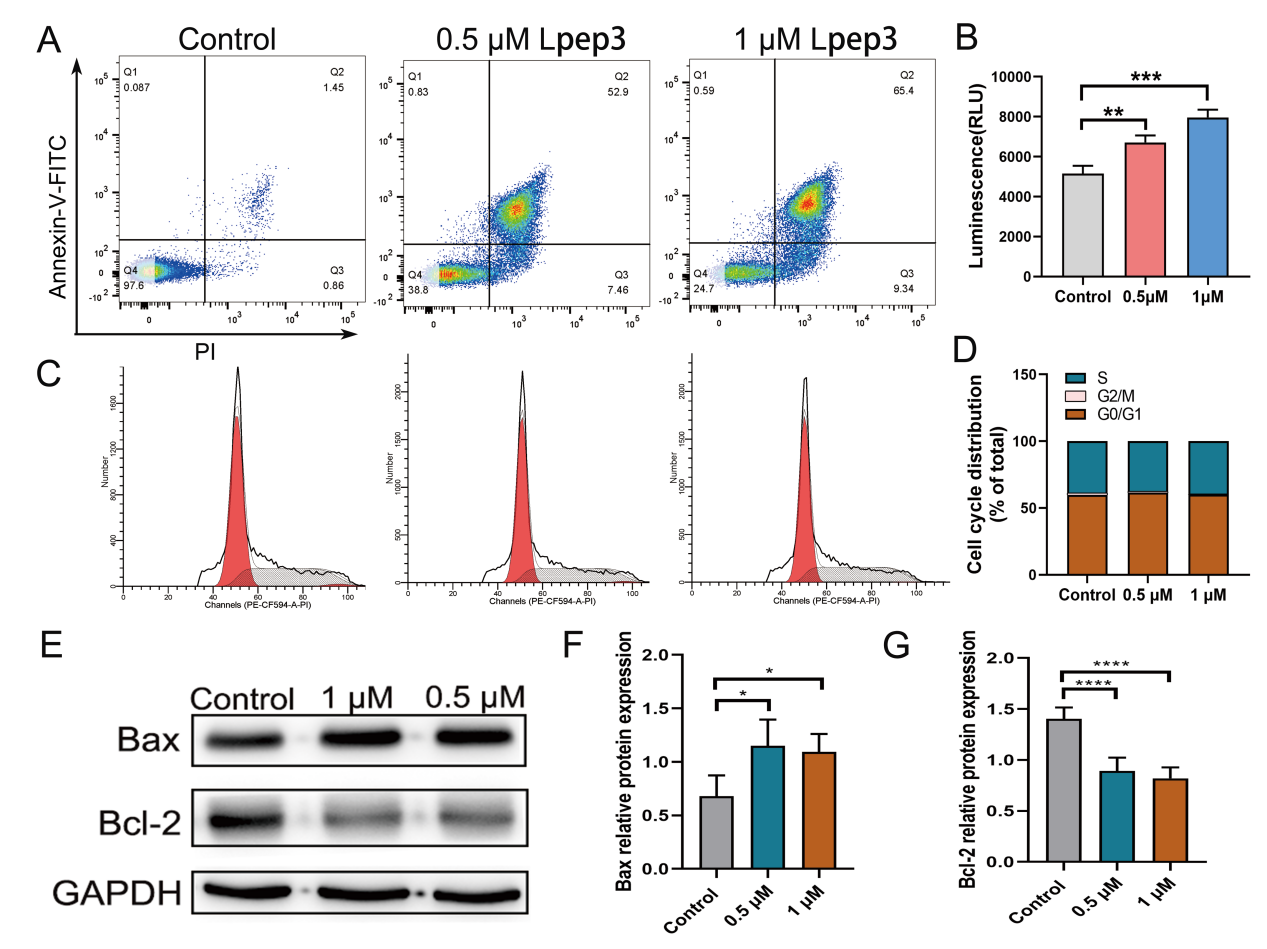
Lpep3 induces apoptosis but has no effect on cell cycle arrest in MV-4-11 cells. (**A**) Apoptosis analysis of MV-4-11 cells treated with 0.5 µM and 1 µM Lpep3 for 24 h, followed by staining with Annexin-V/PI. (**B**) ATP content analysis. (**C**) Cell cycle analysis of MV-4-11 cells treated with 0.5 µM and 1 μM Lpep3 for 24 h by flow cytometry. The red area on the left represents the G0/G1 phase, the red area on the right represents the G2/M phase, and the shaded area is the S phase. (**D**). Quantitative results of cell cycle distribution. (**E**–**G**) Lpep3 decreases the protein expression of Bcl-2 and increases the protein expression of Bax. * *p* < 0.05, ** *p* < 0.01, *** *p* < 0.001, **** *p* < 0.001.

Lpep3 was able to induce cell death through direct and rapid membrane lysis, particularly at higher concentrations. To further explore its potential role in apoptosis and proliferation, cell cycle analysis and Annexin V/PI staining were performed. The results revealed that in Lpep3 treatment, there was increased apoptosis in MV-4-11 cells in a dose-dependent manner compared with untreated controls (Figure 4A). In addition, Lpep3 treatment led to decreased ATP release, implying altered mitochondrial membrane permeability (Figure 4B). However, Lpep3 had no significant effect on cell cycle distribution, suggesting that it does not suppress leukemia cell growth by regulating cell cycle progression (Figure 4C,D). Western blotting analysis demonstrated that Lpep3 markedly upregulated pro-apoptotic protein Bax while downregulating anti-apoptotic Bcl-2 protein expression, indicating caspase activation and apoptosis execution (Figure 4E–G) (Original western blots can be found at Supplementary Materials). Consistently, RT-qPCR assays confirmed that the transcription levels of pro-apoptotic genes (Bax, Casp8, and Casp9) were significantly elevated, whereas those of anti-apoptotic genes (such as Bcl-2 and Bcl2l1) were reduced (Figure S3). Together, these findings suggest that, in addition to direct membrane lysis, Lpep3 promotes apoptosis of MV-4-11 cells via the mitochondria-dependent Bax/Bcl-2 signaling pathway.

### 3.5. Evaluation of the Anticancer Activity and Safety of Lpep3 In Vivo

**Figure 5:**
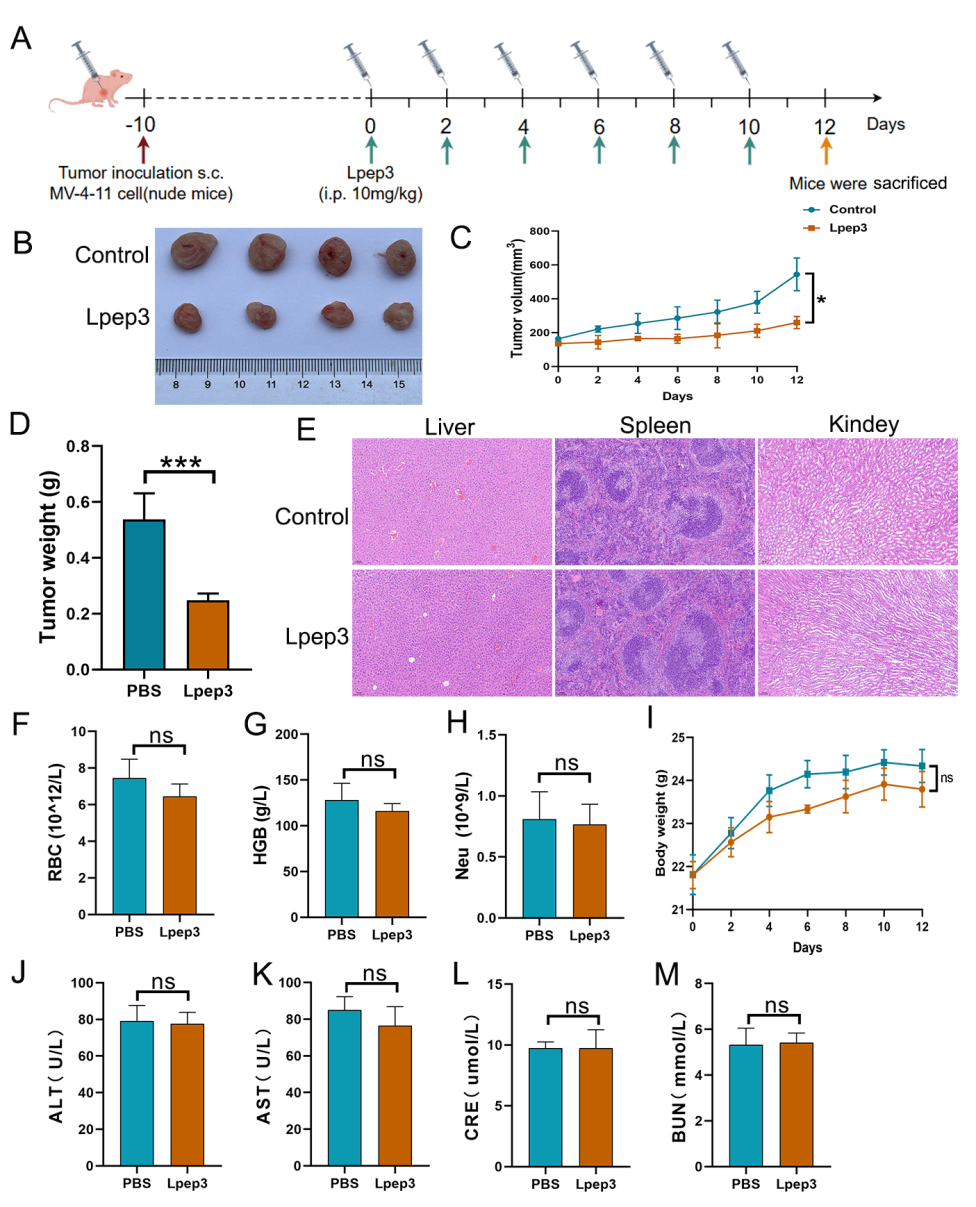
In vivo antitumor activity and safety evaluation of Lpep3. (**A**) Schematic illustration of the schedule for the in vivo therapeutic experiments. (**B**) Images of excised MV-4-11 tumors in nude mice (*n* = 4). (**C**) Growth curves of MV-4-11 tumor formation. (**D**) Quantitative results of tumor weight. (**E**) HE staining between PBS and Lpep3 groups. (**F**–**H**) Data from blood routine examination parameters RBC, HGB, and Neu. (**J**–**M**) The liver and kidney function tests of mice treated with PBS or Lpep3 for 12 days. (**I**) Changes in body weight of BALB/c mice during the study. RBC, red blood cell; Neu, neutrophil count; HGB, hemoglobin; ALT, alanine aminotransferase; AST, aspartate aminotransferase; CRE, creatinine. Data are expressed as mean ± S.E.M., *n* = 4 for each group. Statistical analysis was performed using the *t*-test. * *p* < 0.05, *** *p* < 0.01; ns indicates no significant difference (between the two groups).

Inspired by the cytotoxicity of Lpep3 in vitro, the anticancer activity and safety of Lpep3 in vivo were evaluated. The treatment schedule is shown in Figure 5A. Approximately 10 days after inoculation (when tumor volumes reached 100 to 150 mm^3^), male nude mice bearing tumors were randomly divided into two groups and intraperitoneally injected with PBS and 10 mg/kg Lpep3 (50 µL) every other day, for a total of six injections. As shown in Figure 5B, after the tumors were removed, it could be seen that the tumor volumes in the Lpep3 group were much smaller than those in the control group. By growth curves of MV-4-11 tumors in the mice, it was observed that the tumors in the PBS-treated group continued to grow rapidly, while the tumor volumes in the Lpep3-treated mice showed no obvious increase during the treatment period (Figure 5C). In addition, there was a significant difference in tumor weight between the two groups (Figure 5D).

Subsequently, a systematic in vivo biosafety evaluation was conducted. Blood and tissue samples were collected for the detection of RBC function parameters; liver, spleen, and kidney function indices; and H&E staining. The results showed that there was no obvious difference in the body weight and blood routine indices, including the RBC, HGB, and Neu parameters (Figure 5F–I). The liver and kidney function indices (AST, ALT, UA, CRE) in the Lpep3 group remained within the normal range (Figure 5J–M). Furthermore, H&E staining of liver, spleen, and kidney showed that, compared with the control group, Lpep3 caused no obvious damage to these organs in the mice (Figure 5E). Taken together, these data suggest that Lpep3 can effectively inhibit tumor growth in vivo without significant toxicity.

## 4. Discussion

Numerous previous studies have shown that scorpion-derived peptides can reduce or kill cancer cells through vesicle-mediated internalization or pore formation in the cytoplasmic membrane, a mechanism similar to that of antimicrobial peptides [25]. BmKn2, an antimicrobial peptide isolated from the scorpion M. martensii, mediates apoptosis via the p53-dependent intrinsic apoptotic pathway, demonstrating great potential as a promising therapeutic agent for oral cancer [26]. Similarly, TsAP-2, an antimicrobial peptide cloned from the Brazilian yellow scorpion Tityus serrulatus, can inhibit the growth of H157, H838, MCF-7, PC3, and U251-MG cells [27]. However, evidence suggests a symbiotic relationship between bacteria and certain tumors [28]. In this context, anticancer peptides (ACPs) with dual antibacterial and anticancer properties may offer distinct advantages, thus emerging as a novel therapeutic option for cancer patients, as exemplified by Cecropins [29], Magainin 2 [30], Melittin [31], Lactoferricin [32], and LL-37 [33]. Additionally, a small number of antimicrobial peptides (AMPs) have already entered the clinical phase for cancer treatment [34]. The peptide identified in this study, Lpep3, is a toxin-derived polypeptide that was previously reported as an antimicrobial peptide. However, the present study reveals its anticancer activity against various cancer cell lines, with the strongest potency observed in leukemia cell lines, including HL-60, MV-4-11, K562, and Jurkat. The average IC_50_ value of Lpep3 in leukemia cell lines is approximately 0.5 μM, which is about 10-fold lower than that in normal cells. These findings indicate that antimicrobial peptides from scorpion venom hold significant potential for development as novel anticancer candidates for clinical applications.

Studies have shown that most anticancer peptides (ACPs) adopt an α-helical conformation. Similarly, Lpep3 in the present study exhibits a complete α-helical conformation, which lays a structural foundation for its remarkable anticancer activity. Previous research studies have reported multiple similar toxin peptides. For example, magainins— toxin polypeptides isolated from African clawed frog Xenopus laevis—and their derivatives exhibit a classic α-helical structure and exert antitumor activity against the human lung cancer cell line A549 as well as various human bladder cancer cell lines, with effective concentrations 5–10 times higher than those required for normal cells [35]. Short peptides isolated from the Australian frogs Litoria raniformis and Litoria citropa are composed of 13 and 16 amino acid residues, respectively. They possess an α-helical structure with distinct hydrophobic and hydrophilic regions, displaying broad-spectrum antitumor activity against multiple human cancer cell lines without significant hemolytic effects on red blood cells [36,37].

In addition to directly disrupting cancer cell membranes, the mechanisms of action of anticancer peptides (ACPs) can induce cancer cell death through several other non-membrane-lytic pathways, such as angiogenesis inhibition, tumor cell apoptosis induction, key cellular protein targeting, or immune cell recruitment. For example, ACPs, including BuforinIIb and LL-37, can activate apoptotic signaling pathways by inducing apoptosis and disrupting mitochondrial membranes to release cytochrome C, which in turn activates the caspase cascade and triggers a series of proteolytic reactions, leading to cell disintegration [38,39]. Tachyplesin I, rAGAP, and WP1 inhibit cell proliferation by arresting cancer cells at the G0, G1, or S phase of the cell cycle [40,41,42]. Kahalalide F (KF) induces the death of cancer cells (e.g., melanoma, colon cancer, and breast cancer cells) by disrupting lysosomal membranes and releasing their contents [43]. Modified ACPs, such as PV-S4 and RR-S4, can induce cancer cell death by directly inhibiting DNA synthesis [44]. Studies have shown that activating tumor immunity and regulating the body’s immune response to exert antitumor effects has become a research hotspot in cancer immunotherapy, such as LTX-315, HN-1, and GE33 [45,46,47]. Research indicates that the bee venom peptide Melittin increases Ca^2+^ influx and binds to mitochondria through electrostatic interactions, leading to the leakage of mitochondrial contents, altered cell membrane permeability, and subsequent cancer cell death [48]. In our study, the integrated analysis of lactate dehydrogenase (LDH) release assays and flow cytometry-based apoptosis detection results supports that Lpep3 exerts a concentration- and time-dependent dual anticancer mechanism. Specifically, Lpep3 induces rapid membrane rupture in target cells while simultaneously activating the Bax/Bcl-2-related regulatory axis to trigger apoptotic pathways. This synergistic and multi-targeted mechanism positions Lpep3 as a promising candidate for the treatment of refractory or relapsed leukemia, which typically exhibits resistance to conventional chemotherapeutic regimens.

## 5. Conclusions

In this study, we report for the first time that Lpep3, a peptide with known antimicrobial activity, exhibits broad-spectrum anticancer activity. In in vitro experiments, compared with other solid tumor and hematological tumor cells, Lpep3 selectively exerts a significant inhibitory effect on leukemia cells. A series of experiments demonstrated that Lpep3 can rapidly disrupt cell membranes, activate the Bax/Bcl-2-related apoptotic signaling pathway, and thereby promote cell apoptosis. In vivo experiments showed that this peptide not only has good biosafety but also significantly inhibits the growth of tumor cells in mice. In conclusion, the selective anti-leukemia activity of Lpep3 is coordinately mediated by membrane lysis and apoptosis. This dual mechanism makes Lpep3 a promising candidate for the development of effective and selective anti-leukemia drugs.

## Figures and Tables

**Table 1:** Primer pairs for RT-PCR experiments.

Gene	Forward Primer	Reverse Primer
*Gapdh*	TGCACCACCAACTGCTTAGC	GGCATGGACTGTGGTCATGAG
*Bax*	TGAAGACAGGGGCCTTTTTG	AATTCGCCGGAGACACTCG
*Bcl-2*	TCGCCCTGTGGATGACTGAG	CAGAGTCTTCAGAGACAGCCAGGA
*Bcl2l1*	CAGAGCTTTGAACAGGTAG	GCTCTCGGGTGCTGTATTG
*Casp8*	AGAAGAGGGTCATCCTGGGAGA	TCAGGACTTCCTTCAAGGCTGC
*Casp9*	CTCAGACCAGAGATTCGCAAAC	GCATTTCCCCTCAAACTCTCAA

**Table 2:** Sequences, species, and structure of 7 candidate peptides.

Peptide	Peptide Sequence	Species	Length	Average Prediction Score (*n* = 5)	Structure
Lpep1	LNFKALAALAKKIL	*Polistes rothneyi*	14	0.94	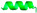
Lpep2	FLPLILRKIVTAL	*Vespa crabro*	13	0.98	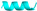
Lpep3	FFGSLLSLGSKLLPSVFKLFQRKKE	*Centruroides suffusus*	25	0.93	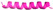
Lpep4	GLFDIAKKVIGVIGSL	*Ranoidea raniformis*	16	0.93	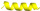
Lpep5	GLWSKIKEAAKTAGKAAMGFVNEMV	*Phyllomedusa trinitatis*	25	0.91	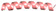
Lpep6	FLPIIGKLLSGLL	*Rana cascadae*	13	0.99	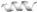
Lpep7	FVQWFSKFLGRIL	*Rana temporaria*	13	0.97	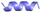

## Data Availability

The original contributions presented in this study are included in the article/Supplementary Material. Further inquiries can be directed to the corresponding authors.

## Supplementary Materials

The following supporting information can be downloaded at: https://www.mdpi.com/article/10.3390/biom15121751/s1. Figure S1: The trends of inhibition rates of 6 candidate peptides against 12 mouse-derived cancer cell lines at 5 μM, 10 μM, and 20 μM; Figure S2: The IC_50_ values of Lpep3 in Raji cells and hemolytic activity of Lpep3 at different concentrations; Figure S3: Real-time RT-PCR was used to measure apoptosis-related gene mRNA expression. Figure S4: Western Blotting Figure. File S1: The purity or identity characterization of the synthesized peptides.
