# Supplementary material for: A Scorpion Peptide Exerts Selective Anti-Leukemia Effects Through Disrupting Cell Membranes and Triggering Bax/Bcl-2-Related Apoptosis Pathway
Source: Biomolecules. 2025 Dec 18;15(12):1751. doi: 10.3390/biom15121751 (PMC12730667; doi:10.3390/biom15121751)
Supplement: Supplementary file 1 [file biomolecules-15-01751-s001.zip › supplement meterials File S1/HPLC report/FCL-NJP93901 Lpep1 1263335 HPLC.pdf]

HPLC REPORT

Sample:	FCL-NJP93901 Lpep1 LL-14	Analyzed date:	2025-5-26
Analyst:	WGJ	Reconstitution:	H2O:ACN=4:1
Lot. No.:	P250521-WY1263335		
Column:	COSMOSIL Packed Column, 4.6*250mm, 5µm		
Solvent A	A: 0.1% Trifluoroacetic Acid in 100% Acetonitrile		
Solvent B	B: 0.1% Trifluoroacetic Acid in 100% Water		

Gradient:

	A	B
0.0min	30%	70%
25.0min	55%	45%
25.1min	100%	0%
30.0min	Stop	

Volume: 5µl
Wavelength: 220nm
Flow rate: 1.0ml/min

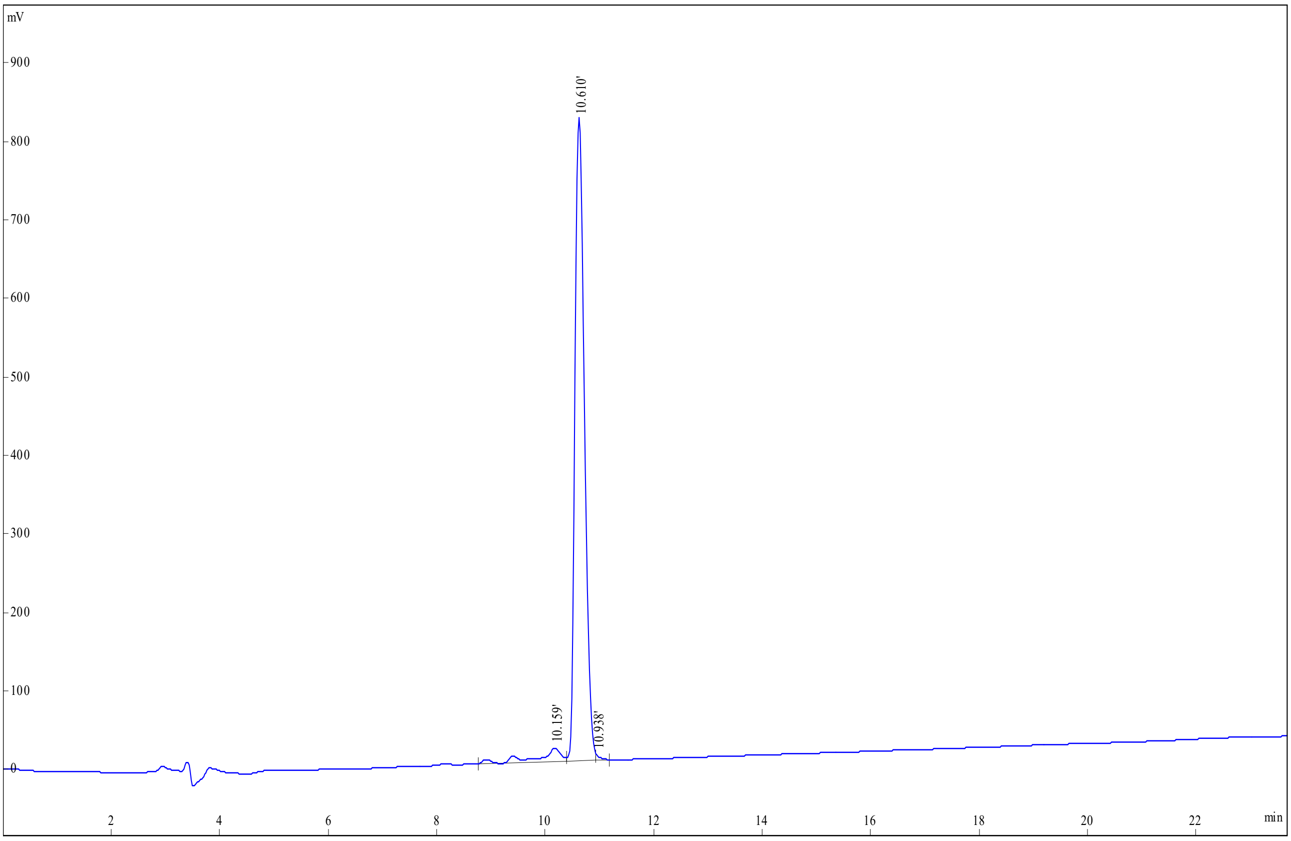

Peak	Time	Conc.	Area	Height
1	10.159	4.35	455281	16569
2	10.610	95.4	9984130	821210
3	10.938	0.2546	26648	5579
Total		100	10466059	843358
