# Supplementary material for: A Scorpion Peptide Exerts Selective Anti-Leukemia Effects Through Disrupting Cell Membranes and Triggering Bax/Bcl-2-Related Apoptosis Pathway
Source: Biomolecules. 2025 Dec 18;15(12):1751. doi: 10.3390/biom15121751 (PMC12730667; doi:10.3390/biom15121751)
Supplement: Supplementary file 1 [file biomolecules-15-01751-s001.zip › supplement meterials File S1/HPLC report/FCL-NJP93902 Lpep2 1263339 HPLC.pdf]

HPLC REPORT

Sample:	FCL-NJP93902 Lpep2 FL-13	Analyzed date:	2025-5-26
Analyst:	WGJ	Reconstitution:	H20:ACN=3:1
Lot. No.:	P250521-WY1263339		
Column:	4.6×250mm,Agela C18-5		
Solvent A	A: 0.1% Trifluoroacetic Acid in 100% Acetonitrile		
Solvent B	B: 0.1% Trifluoroacetic Acid in 100% Water		

Gradient:

	A	B
0.0min	35%	65%
25.0min	60%	40%
25.1min	100%	0%
30.0min	Stop	

Volume: 5μl
Wavelength: 220nm
Flow rate: 1.0ml/min

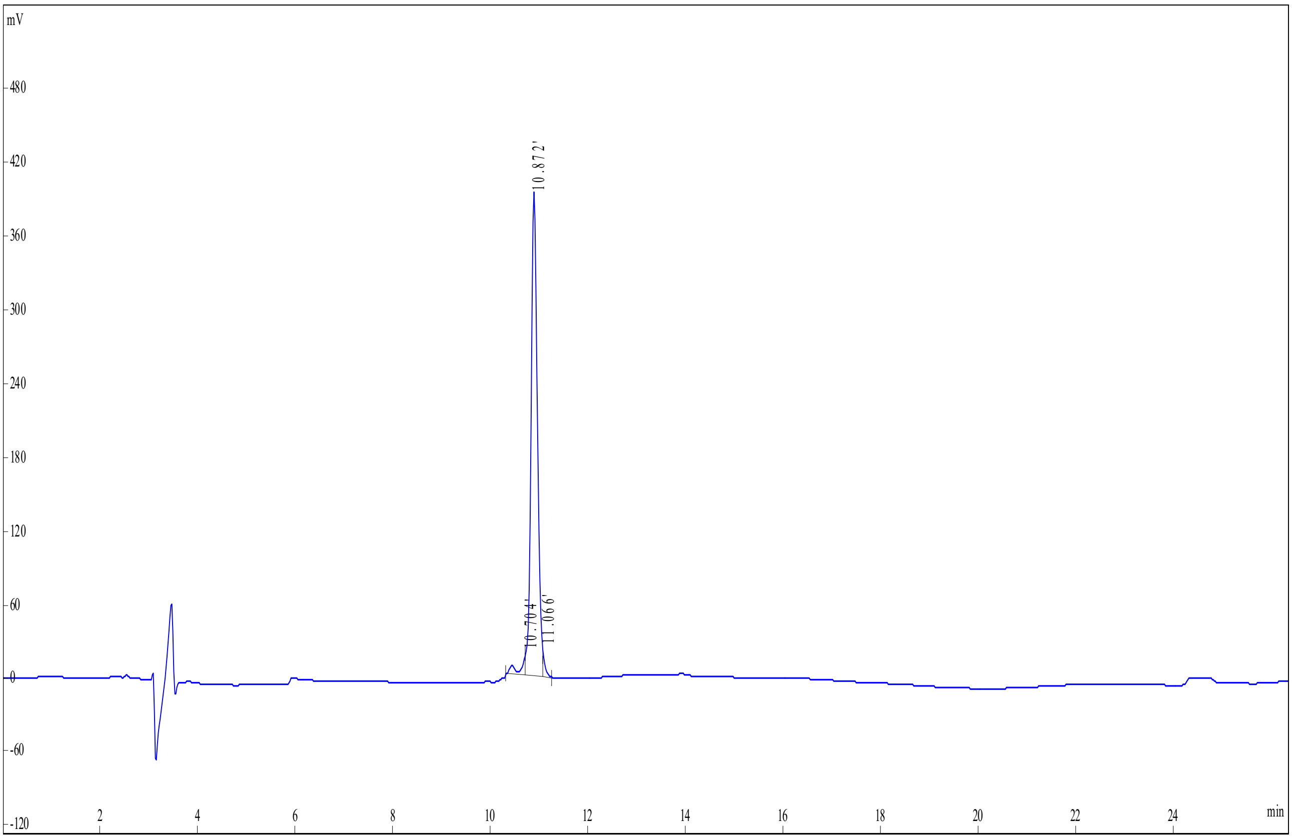

Peak	Time	Conc	Area	Height
1	10.704	3.534	131848	17926
2	10.872	95.18	3551249	395291
3	11.066	1.284	47904	16800
Total		100	3731001	430017
