# Supplementary material for: A Scorpion Peptide Exerts Selective Anti-Leukemia Effects Through Disrupting Cell Membranes and Triggering Bax/Bcl-2-Related Apoptosis Pathway
Source: Biomolecules. 2025 Dec 18;15(12):1751. doi: 10.3390/biom15121751 (PMC12730667; doi:10.3390/biom15121751)
Supplement: Supplementary file 1 [file biomolecules-15-01751-s001.zip › supplement meterials File S1/HPLC report/FCL-NJP93903 Lpep 3 1263340 HPLC.pdf]

HPLC REPORT

Sample: FCL-NJP93903 Lpep 3 FE-25
Analyzed date: 2025-6-4
Analyst: HXH
Reconstitution: 1MG/1ML DMSO
Lot. No.: P250521-WY1263340
Column: Gemini-NX 5μ C18 110A, 4.6*250mm
Solvent A: A: 0.1% Trifluoroacetic Acid in 100% Acetonitrile
Solvent B: B: 0.1% Trifluoroacetic Acid in 100% Water

Gradient:

	A	B
0.0min	35%	65%
25.0min	90%	10%
25.1min	100%	0%
30.0min	Stop	

Volume: 20μl
Wavelength: 220nm
Flow rate: 1.0ml/min

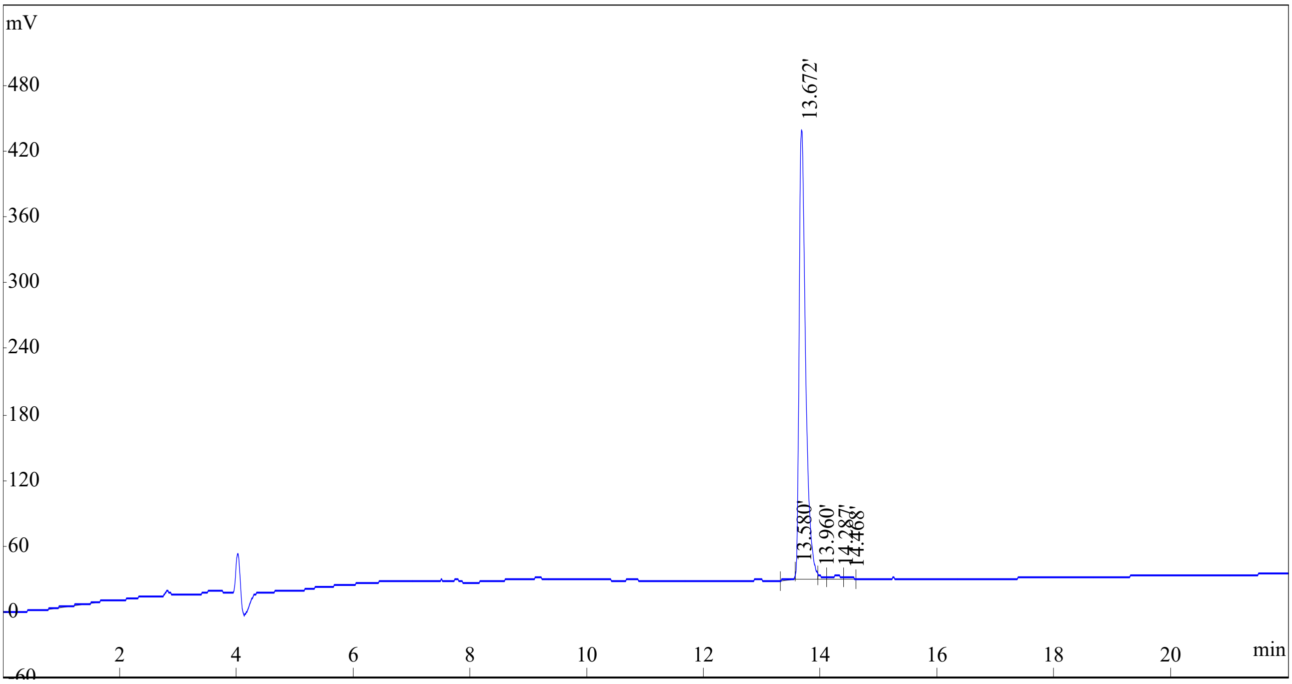

Rank	Time	Conc.	Area	Height
1	13.580	0.3176	9511	7385
2	13.672	97.3562	2915586	409663
3	13.960	0.6570	19677	4233
4	14.287	1.2008	35962	3020
5	14.468	0.4684	14027	1643
Total		100	2994763	425944
