# Supplementary material for: A Scorpion Peptide Exerts Selective Anti-Leukemia Effects Through Disrupting Cell Membranes and Triggering Bax/Bcl-2-Related Apoptosis Pathway
Source: Biomolecules. 2025 Dec 18;15(12):1751. doi: 10.3390/biom15121751 (PMC12730667; doi:10.3390/biom15121751)
Supplement: Supplementary file 1 [file biomolecules-15-01751-s001.zip › supplement meterials File S1/HPLC report/FCL-NJP93906 Lpep5 1263342 HPLC.pdf]

HPLC REPORT

Sample:	FCL-NJP93906 Lpep5 GV-25		Analyzed date:	2025-6-5
Analyst:	HXH		Reconstitution:	1MG/1ML DMSO
Lot. No.:	P250521-WY1263342			
Column:	Gemini-NX 5µ C18 110A, 4.6*250mm			
Solvent A	A: 0.1% Trifluoroacetic Acid in 100% Acetonitrile			
Solvent B	B: 0.1% Trifluoroacetic Acid in 100% Water			

Gradient:		A	B
	0.0min	25%	75%
	25.0min	85%	15%
	25.1min	100%	0%
	30.0min	Stop	

Volume: 20µl

Wavelength: 220nm

Flow rate: 1.0ml/min

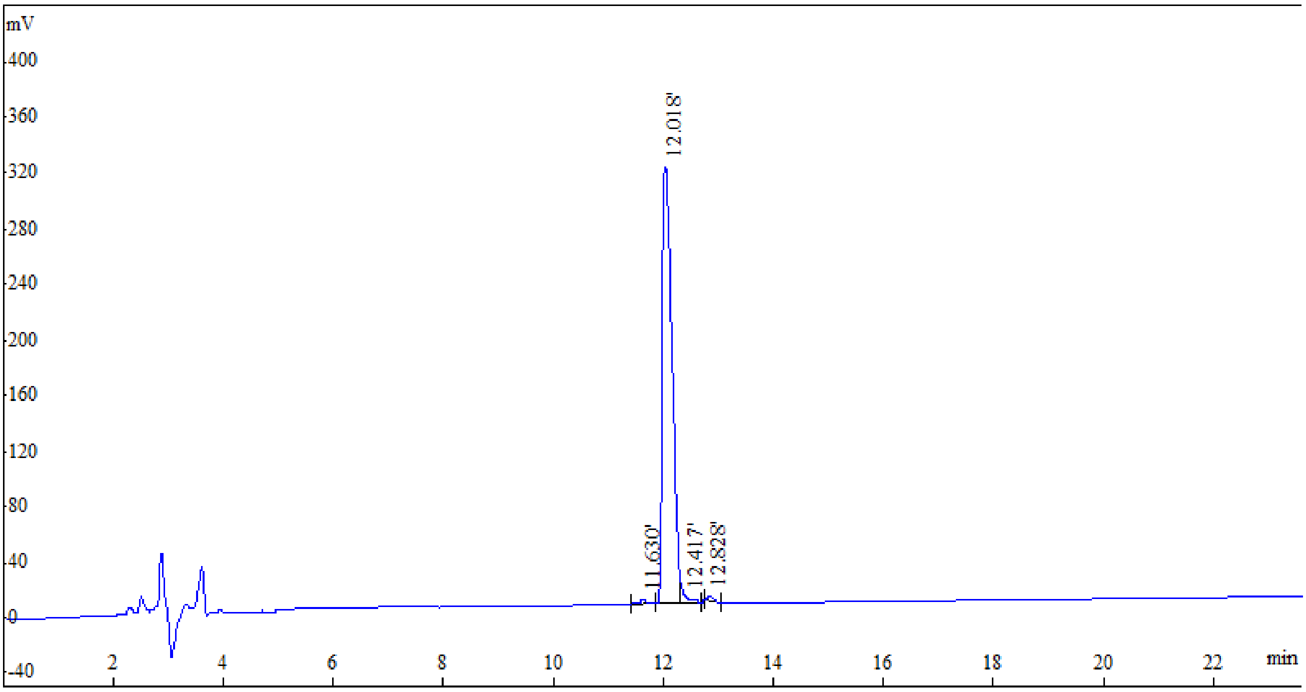

Rank	Time	Conc.	Area	Height
1	11.630	0.5347	19908	1594
2	12.018	97.6551	3635821	313011
3	12.417	1.1522	42898	2124
4	12.828	0.6580	24497	3724
Total		100	3723124	320453
