# Supplementary material for: A Scorpion Peptide Exerts Selective Anti-Leukemia Effects Through Disrupting Cell Membranes and Triggering Bax/Bcl-2-Related Apoptosis Pathway
Source: Biomolecules. 2025 Dec 18;15(12):1751. doi: 10.3390/biom15121751 (PMC12730667; doi:10.3390/biom15121751)
Supplement: Supplementary file 1 [file biomolecules-15-01751-s001.zip › supplement meterials File S1/HPLC report/FCL-NJP93907 Lpep6 569027 HPLC.pdf]

HPLC REPORT

Sample:	FCL-NJP93907 Lpep6 FL-13	Analyzed date:	2025-06-05
Analyst:	LJJ	Reconstitution:	1mg/0.5ml H2O+0.1mlACN
Lot. No.:	P250521-WY569027		
Column:	4.6×250mm,ChromCore 120 C18 5u		
Solvent A	A: 0.1% Trifluoroacetic Acid in 100% Acetonitrile		
Solvent B	B: 0.1% Trifluoroacetic Acid in 100% Water		

Gradient:

	A	B
0.0min	44%	56%
25.0min	69%	31%
25.1min	100%	0%
30.0min	Stop	

Volume: 5μl

Wavelength: 220nm

Flow rate: 1.0ml/min

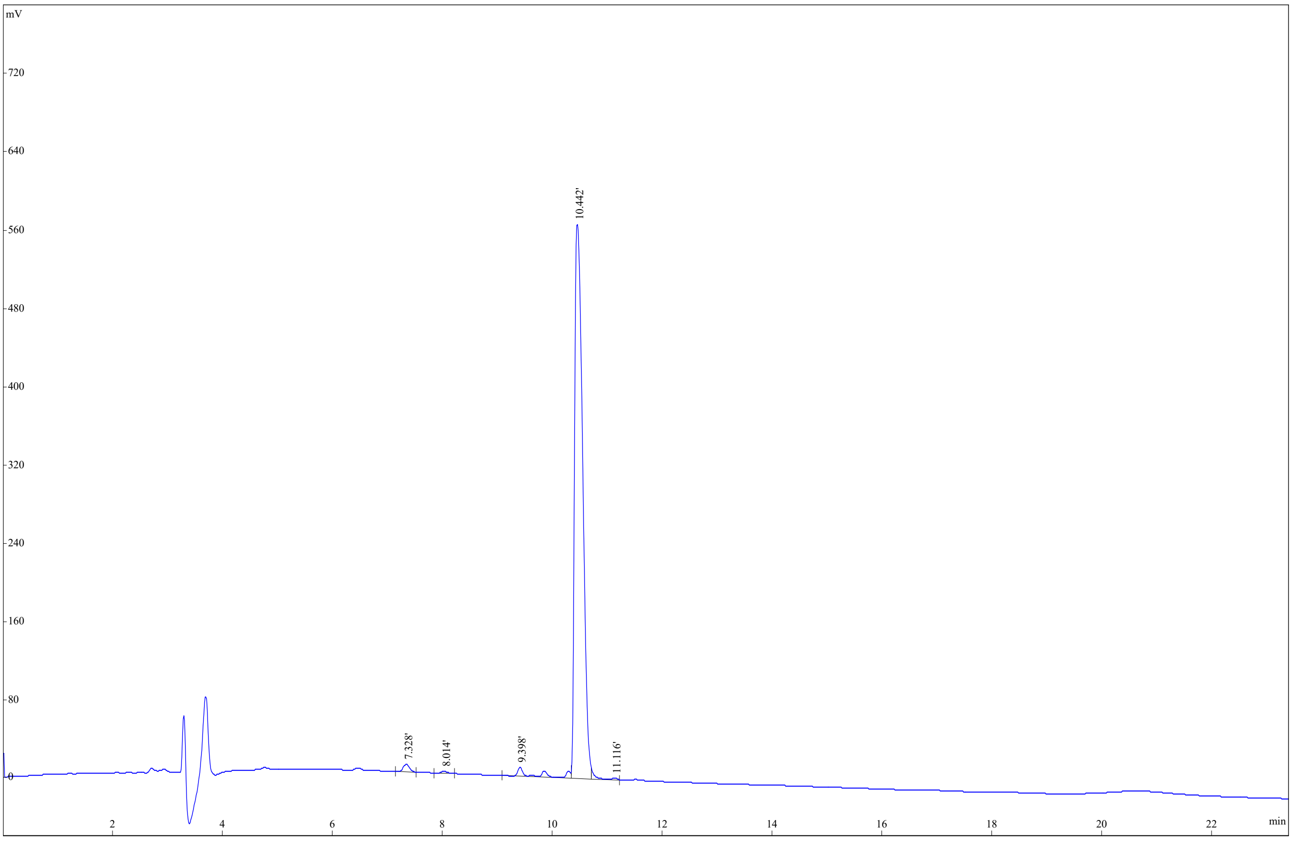

Rank	Time	Conc.	Area	Height
1	7.328	0.9529	53713	7261
2	8.014	0.2618	14760	2261
3	9.398	2.106	118726	8426
4	10.442	96.1	5416686	566355
5	11.116	0.5813	32766	1638
Total		100	5636651	585941
