# Supplementary material for: A Scorpion Peptide Exerts Selective Anti-Leukemia Effects Through Disrupting Cell Membranes and Triggering Bax/Bcl-2-Related Apoptosis Pathway
Source: Biomolecules. 2025 Dec 18;15(12):1751. doi: 10.3390/biom15121751 (PMC12730667; doi:10.3390/biom15121751)
Supplement: Supplementary file 1 [file biomolecules-15-01751-s001.zip › supplement meterials File S1/HPLC report/FCL-NJP93908 Lpep7 053835 HPLC.pdf]

# HPLC REPORT

| | | | |
|---|---|---|---|
| Sample: | FCL-NJP93908 Lpep7 FL-13 | Analyzed date: | 2025-06-05 |
| Analyst: | LJJ | Reconstitution: | 1mg/0.5ml H2O+0.1mlACN |
| Lot. No.: | P250521-WY053835 | | |
| Column: | 4.6×250mm,ChromCore 120 C18 5u | | |
| Solvent A | A: 0.1% Trifluoroacetic Acid in 100% Acetonitrile | | |
| Solvent B | B: 0.1% Trifluoroacetic Acid in 100% Water | | |

Gradient:

| | A | B |
|---|---|---|
| 0.0min | 35% | 65% |
| 25.0min | 60% | 40% |
| 25.1min | 100% | 0% |
| 30.0min | Stop | |

Volume: 5µl

Wavelength: 220nm

Flow rate: 1.0ml/min

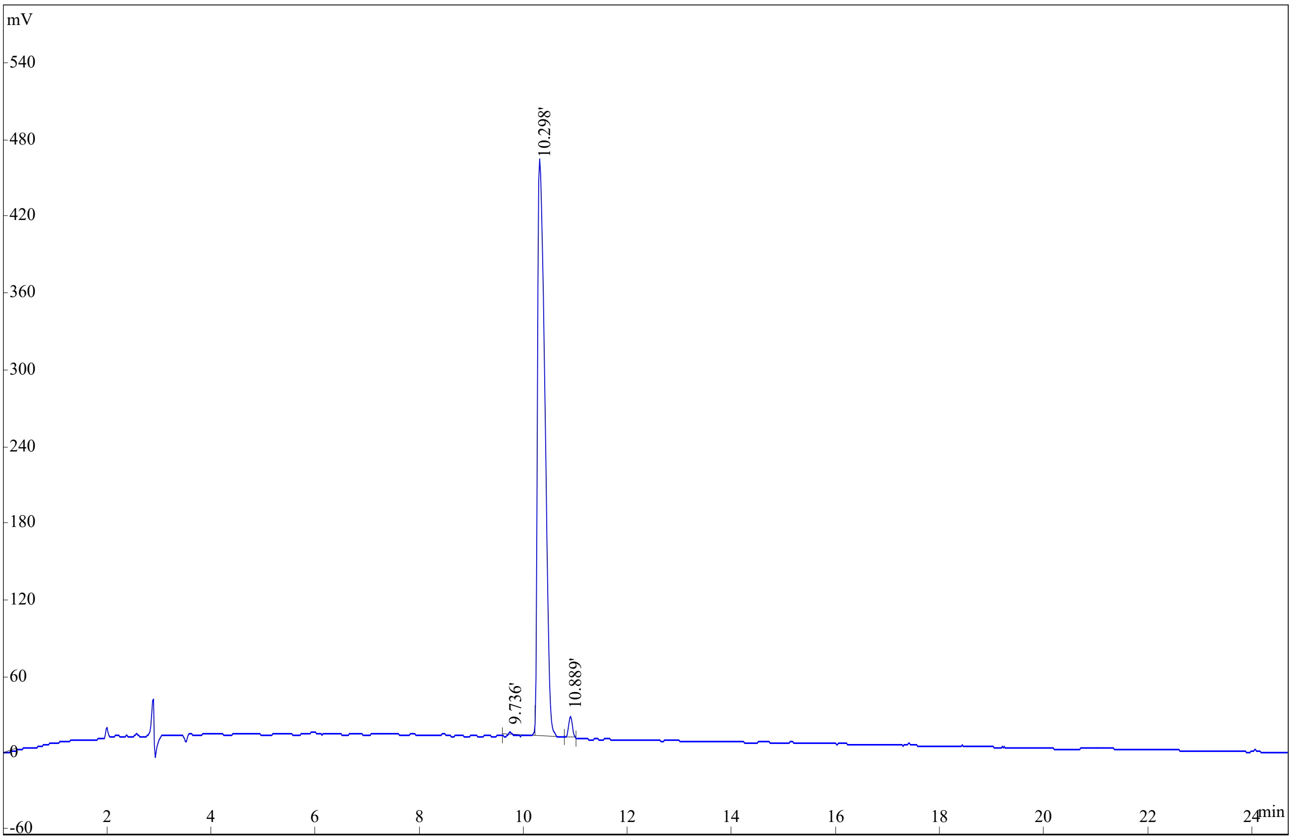


| Rank | Time | Conc. | Area | Height |
|---|---|---|---|---|
| 1 | 9.736 | 1.123 | 51827 | 2976 |
| 2 | 10.298 | 96.9 | 4472136 | 452602 |
| 3 | 10.889 | 1.971 | 90954 | 17411 |
| Total | | 100 | 4614917 | 472989 |
