# Supplementary material for: A Scorpion Peptide Exerts Selective Anti-Leukemia Effects Through Disrupting Cell Membranes and Triggering Bax/Bcl-2-Related Apoptosis Pathway
Source: Biomolecules. 2025 Dec 18;15(12):1751. doi: 10.3390/biom15121751 (PMC12730667; doi:10.3390/biom15121751)
Supplement: Supplementary file 1 [file biomolecules-15-01751-s001.zip › supplement meterials File S1/HPLC report/FCL-NJP93909 Lpep4 1263343 HPLC.pdf]

HPLC REPORT

Sample:	FCL-NJP93909 Lpep8 GL-16	Analyzed date:	2025-6-5
Analyst:	HXH	Reconstitution:	1MG/1ML DMSO
Lot. No.:	P250521-WY1263343		
Column:	Gemini-NX 5μ C18 110A, 4.6*250mm		
Solvent A	A: 0.1% Trifluoroacetic Acid in 100% Acetonitrile		
Solvent B	B: 0.1% Trifluoroacetic Acid in 100% Water		

Gradient:		A	B
	0.0min	40%	60%
	25.0min	90%	10%
	25.1min	100%	0%
	30.0min	Stop	

Volume:	20μl
Wavelength:	220nm
Flow rate:	1.0ml/min

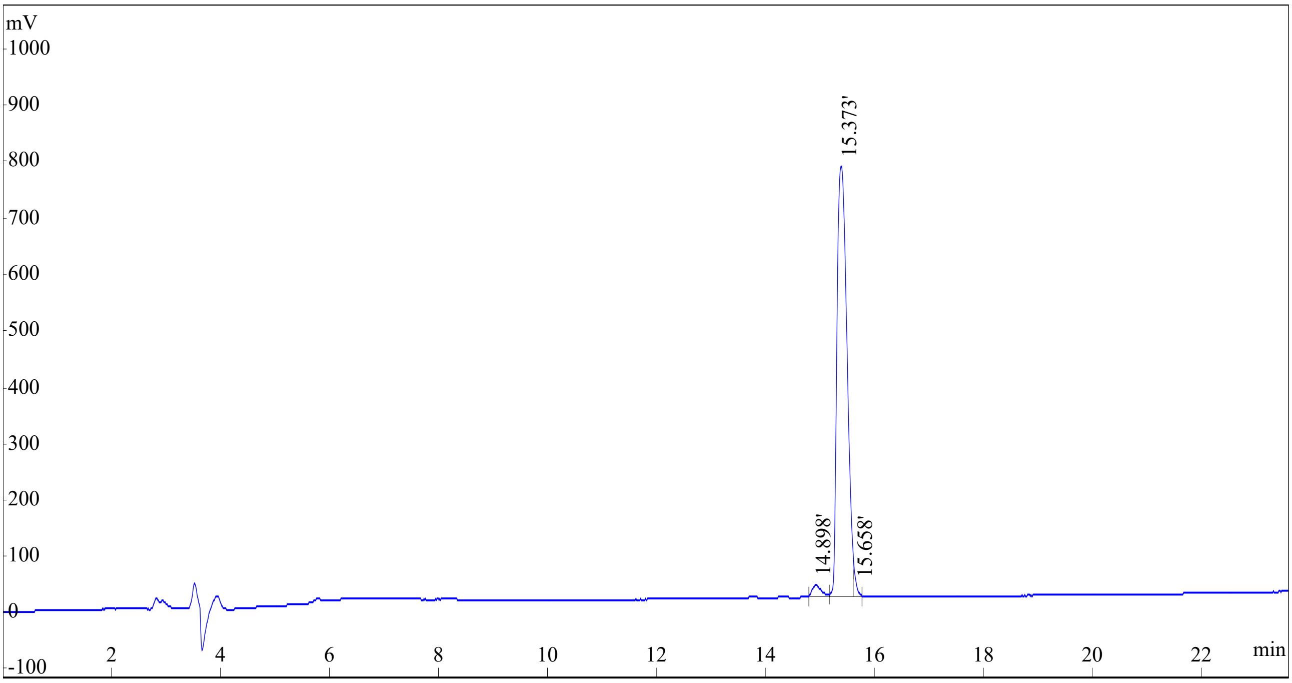

Rank	Time	Conc.	Area	Height
1	14.898	2.0193	192591	18270
2	15.373	96.7513	9227577	760728
3	15.658	1.2294	117255	16554
Total		100	9537423	795552
