# Supplementary material for: A Scorpion Peptide Exerts Selective Anti-Leukemia Effects Through Disrupting Cell Membranes and Triggering Bax/Bcl-2-Related Apoptosis Pathway
Source: Biomolecules. 2025 Dec 18;15(12):1751. doi: 10.3390/biom15121751 (PMC12730667; doi:10.3390/biom15121751)
Supplement: Supplementary file 1 [file biomolecules-15-01751-s001.zip › supplement meterials File S1/MS report/FCL-NJP93908 Lpep7 053835 MS.pdf]

MASS SPECTROMETRY REPORT

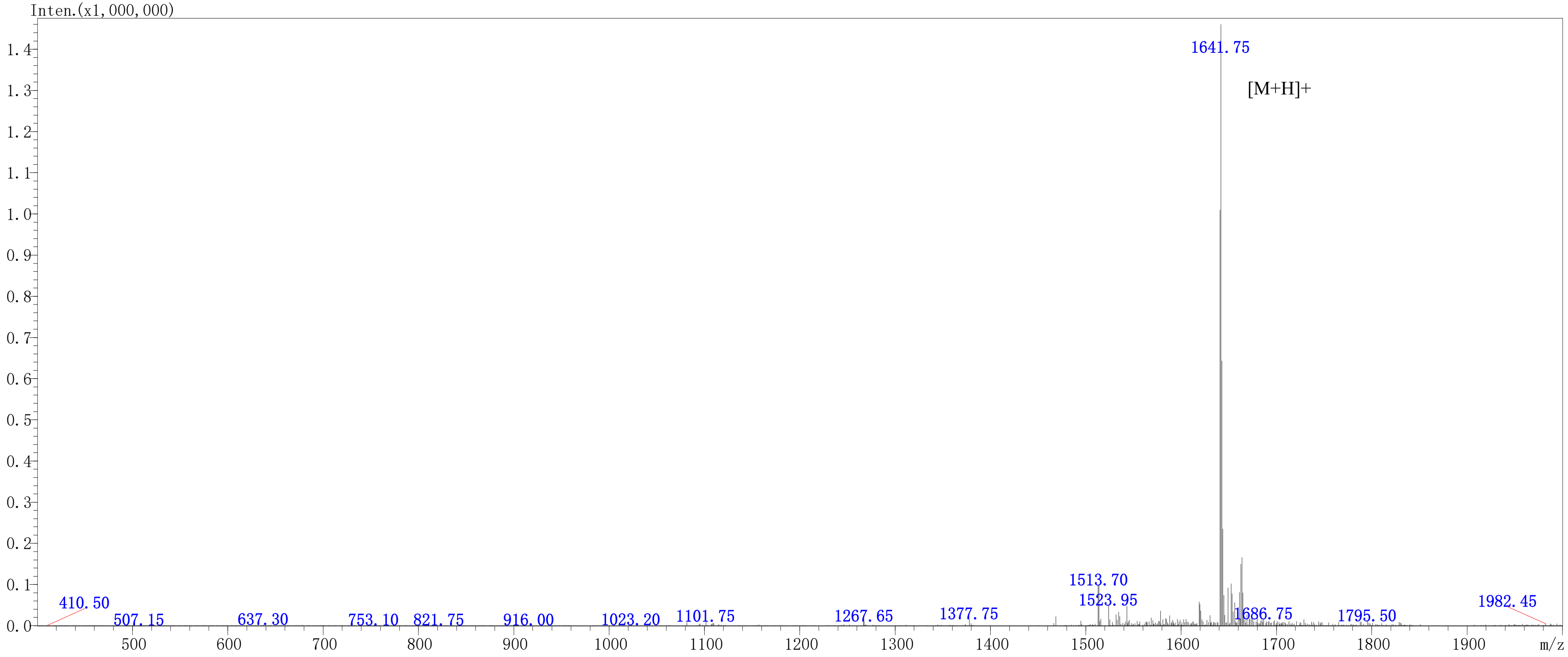

Sample Description		Instrument	SHIMADZU LCMS-2020		
Analyzed date:	2025/6/5	Probe:	ESI	Probe Bias:	+4.5kv
Analyst:	Shen	Nebulizer Gas Flow:	1.5L/min	Detector:	1.2kv
Sample:	FCL-NJP93908 Lpep7 FL-13	CDL:	-20.0v	T. Flow:	0.2ml/min
M.W.:	1640.97	CDL Temp.:	250 °C	B. Conc.:	50%H2O/50%ACN
Lot. No.:	P250521-WY053835	Block Temp.:	400 °C		
