# Supplementary figures and images for: A Scorpion Peptide Exerts Selective Anti-Leukemia Effects Through Disrupting Cell Membranes and Triggering Bax/Bcl-2-Related Apoptosis Pathway
Source: Biomolecules. 2025 Dec 18;15(12):1751. doi: 10.3390/biom15121751 (PMC12730667; doi:10.3390/biom15121751)

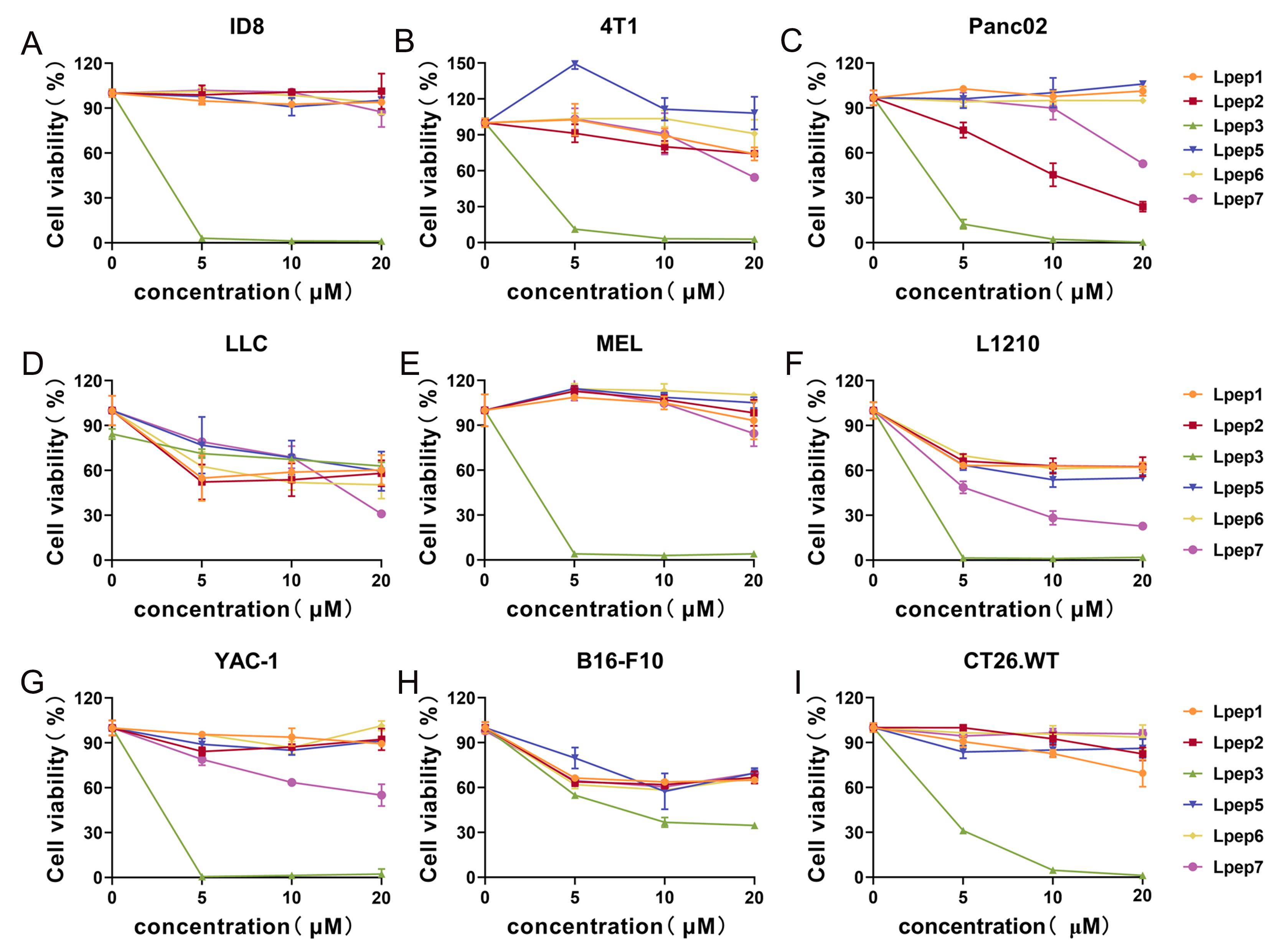

Supplement: Supplementary file 1 [file biomolecules-15-01751-s001.zip › Supplement Figure S1.tif]

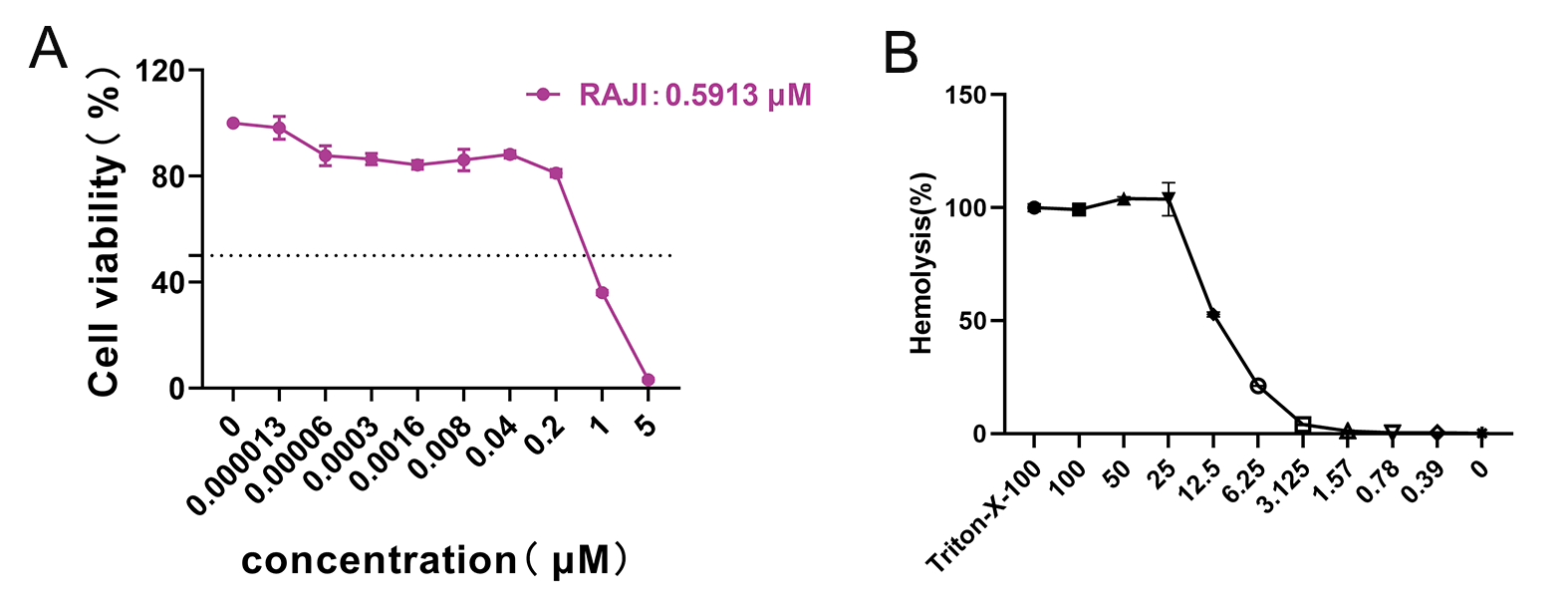

Supplement: Supplementary file 1 [file biomolecules-15-01751-s001.zip › Supplement Figure S2.tif]

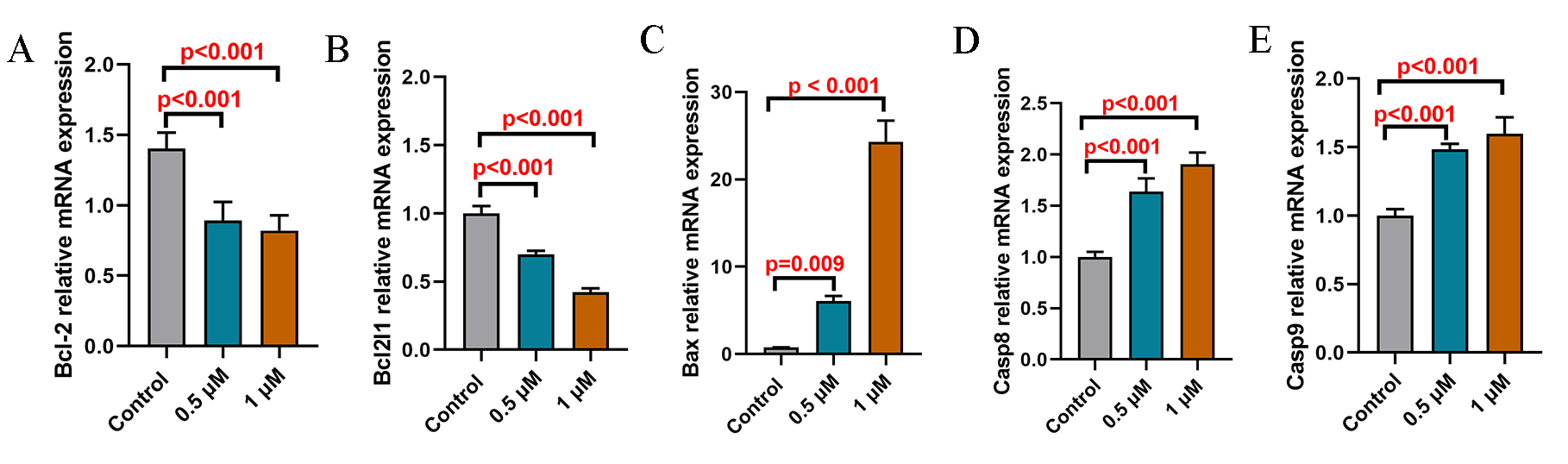

Supplement: Supplementary file 1 [file biomolecules-15-01751-s001.zip › Supplement Figure S3.tif]

**Figure 4**

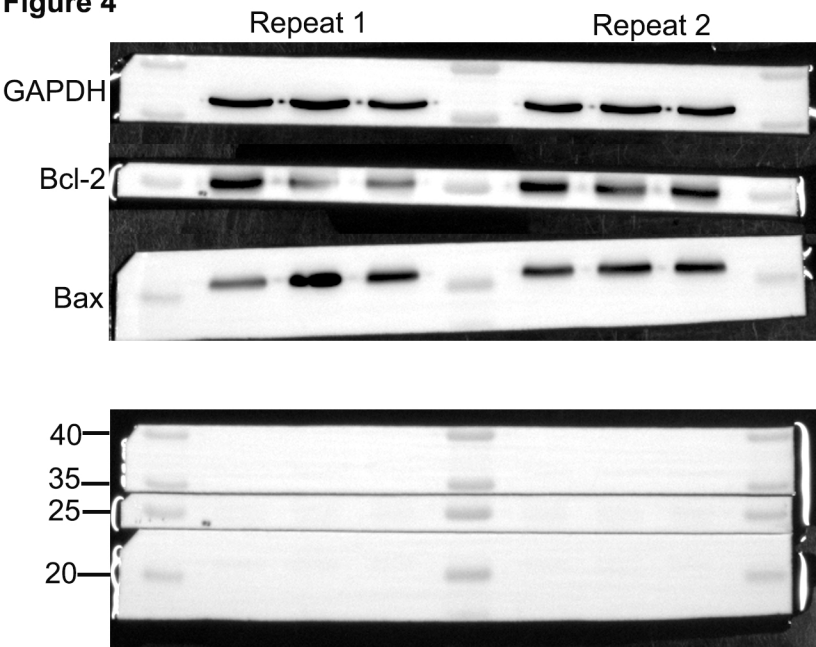

Figure 4-Bax Repeat 3

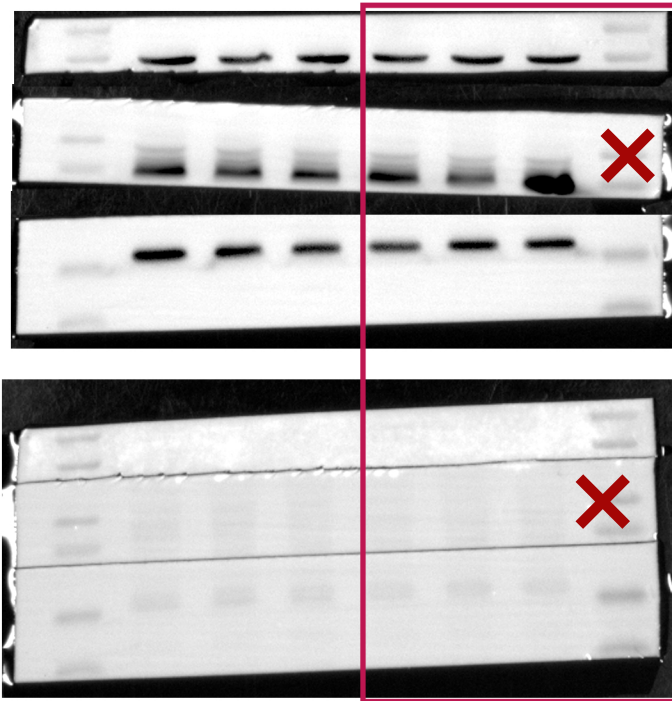

Figure 4-Bcl-2 Repeat 3

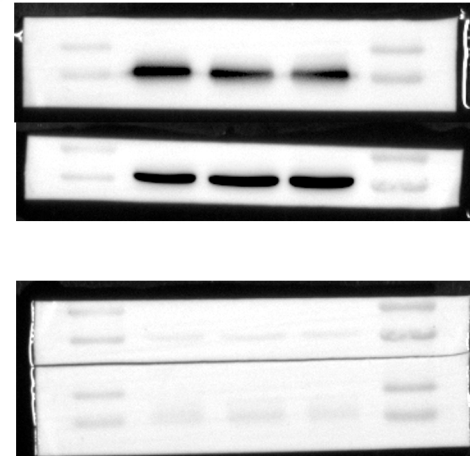

Supplement: Supplementary file 1 [file biomolecules-15-01751-s001.zip › Supplement Figure S4-original_images.pdf]
